# Reliability and validity of the Caregiver Reported Early Development Instruments (CREDI) in impoverished regions of China

**DOI:** 10.1186/s12887-020-02367-4

**Published:** 2020-10-12

**Authors:** Ying Li, Lei Tang, Yu Bai, Shuhang Zhao, Yaojiang Shi

**Affiliations:** 1 Center for Experimental Economics in Education, Shaanxi Normal University, Xi’an, 710119 Shaanxi Province China; 2 School of Economics, Minzu University of China, China Institute for Vitalizing Border Areas and Enriching the People, Beijing, 100081 China

**Keywords:** Early childhood development, CREDI, Reliability, Validity, Impoverished regions of China

## Abstract

**Background:**

There is a great need in low- and middle- income countries for sound qualitative and monitoring tools assessing early childhood development outcomes. Although there are many instruments to measure the developmental status of infants and toddlers, their use in large scale studies is still limited because of high costs in both time and money. The Caregiver Reported Early Development Instruments (CREDI), however, were designed to serve as a population-level measure of early childhood development for children from birth to age three, and have been used in 17 low- and middle-income countries. This study aimed to examine the reliability and validity of the CREDI in China, which is still unknown.

**Methods:**

The CREDI and the ASQ-3 was administered to a sample of 946 children aged 5–36 months from urban and rural communities, in which 248 children was administered with Bayley-III.

**Results:**

The internal consistency of the CREDI was high, which indicates that the scale internal consistency reliability is quite good. The results also indicated that the concurrent validity of the CREDI with the Bayley-III scale was high in general. Ordinary least squares regression showed that the CREDI is highly consistent with previous widely used instruments in some key predictors (such as the home stimulation) of early childhood development level.

**Conclusions:**

All the results in the current study indicate that the CREDI may be considered an appropriate instrument to measure early childhood development status on a large scale in impoverished regions of China.

## Background

It is widely known that the emotional, social, and cognitive skills that emerge in early childhood are important prerequisites for success in school, employment, and potential income in the later stages of an individual’s life [11, 12, 29]. The period from birth to 3 years is also the stage when development is most rapid and children at this stage begin reaching basic development milestones. Therefore, early childhood is very sensitive to environmental effects and is also a period suitable for interventions that alleviate effects of external risk factors [6, 17, 34–36].

Early childhood development (henceforth, “ECD”) has been recognized by governments and Non-Governmental Organizations as a window of opportunity to improve the level of individual development and the social and economic well-being of society as a whole [6]. Under this context, continuous monitoring of ECD outcomes using culturally and developmentally appropriate instruments can provide useful information for developing more effective intervention strategies [8]. Moreover, population-level measurement of ECD is necessary to improve ECD outcomes and reduce developmental inequality through national, regional, and global policies [25].

Although there has been significant progress in supporting and monitoring ECD and developing instruments assessing ECD, there are few effective and reliable instruments available to assess children’s early development status at a large scale in different cultural environments [25]. As Kelly et al. [16] pointed out, children have different ways and times to acquire motor, cognitive, and language skills in different settings. Differences in preschool children’s cognitive and social emotional skills at the national level were found to be related to the country’s socioeconomic and nutritional status [24]. The assessment of ECD in different regions of the world helps us understand commonalities and differences in, and factors contributing to ECD, thus providing useful information for developing more effective intervention strategies.

In summary, population-level measurement and evaluation of ECD is a key issue that needs to be solved in the current era. Unlike individual assessment instruments, population-level measurement tools need to be simple and inexpensive to implement and require cross-cultural comparisons [25]. In this context, a new instrument, the Caregiver Reported Early Development Instruments (CREDI), was developed. The CREDI was designed as a caregiver-reported, cross-culturally comparable, population-level measure of ECD for children under 3 years [25, 26]. The goal of the CREDI is to provide low-cost, large-scale data to facilitate policy interventions and resource allocation, while tracking global progress in alleviating ECD-related disparities around the world [27]. The reliability and validity of the CREDI have been studied and its applicability to the evaluation of ECD levels in low- and middle-income countries has been confirmed [1, 25, 27], but there is still a lack of research about its application in China, and there are still no studies on the application of CREDI in a longer format (henceforth, “long form”). Based on this, this paper introduces and analyzes the application of the CREDI long form in China, with special focus on its reliability and validity, based on survey data from poverty-stricken areas in China.

## Literature review

### Chinese context

In low- and middle-income countries, about 249 million children under the age of five are at risk of poor development, of whom 17.43 million (about 8%) are in China, ranking second in the world [21]. Studies show that concern for ECD in poverty-stricken areas of China is particularly acute [23, 45, 46, 50]. About half of children in poor rural China are at risk for cognitive delays; 52% of children are at risk for language delays, and the risk of delay increases over time [48].

The Chinese government has made many efforts to promote appropriate ECD. At the Central Economic Work Conference in December 2018, “increasing investment in preschool education, early childhood development and vocational education in rural poverty areas” were listed as key tasks. In May 2019, the General Office of the State Council officially issued the “Guiding Opinions on Promoting the Development of Infant and Child Care Services under 3 Years of Age,” and clearly established policies and regulations, standards and norms, and service supply systems to promote the development of childcare services. Childcare services will be implemented in various forms to gradually meet the people’s needs. The Chinese government’s policies reflect its determination to promote ECD and related public service systems. In this context, data collection and evaluation of population-level ECD is particularly urgent. The application of a population-level assessment instrument for ECD has also become an important element in guiding how the Chinese government can effectively implement childcare services policies.

### Existing measures of ECD

Several international instruments have been developed to comprehensively measure ECD, such as the Griffith Mental Development Scales, the Denver Developmental Screening Test, and the Bayley Scales of Infant and Toddler Development, which are direct assessment tools usually done by clinically trained personnel [37] for screening and diagnosis of children with developmental disabilities or delay. Among these measures, the Bayley Scales of Infant and Toddler Development is more widely used in China. Although these individual screening tools have detailed, standard, and practical advantages in obtaining information on a child’s developmental status, there are limitations in providing population-level measurement of ECD because the costs of copyright purchases, adaptation, administration time, and training of the administrators are often relatively high, making them unsuitable for large-scale use [9]. Moreover, this assessment tool directly engages with the child, which may result in measurement errors caused by external factors, such as temperament of the child (e.g. some children might be too shy because of unfamiliarity with the testing environment and the tester) or the ability of children to understand the verbal instructions given.

Therefore, some indirect assessments, which are reported by the caregiver, such as the Early Development Index [15], PRIDI [43], IDELA [30], and the Early Childhood Index [41], may be more suitable for capturing the population’s ECD at this age. Moreover, accessing the data in this way is scalable. However, these instruments are still limited because of the small number of measurement items or their age limits (somewhat concerned with older children rather than 0–3-year-old children). Then the Ages and Stages Questionnaire, third edition (ASQ-3) and the Caregiver Reported Early Childhood Development Instruments (CREDI) were developed for assessing ECD status of infants and toddlers. Compared to direct assessment tools, an instrument reported by caregivers requires less training and testing time, and such an instrument is less likely to be biased by children being unfamiliar with clinical assessments, potential behavior changes with strangers, or not understanding verbal instructions [9, 37]. Previous studies about the reliability and validity of the ASQ-3 and the CREDI will be introduced in detail below.

With respect to the ASQ-3, it is a caregiver-reported measurement that asks caregivers to report different aspects of their children’s behavior to assess development [40]. Although its items have good to acceptable internal consistency [39], previous studies on the validity of ASQ-3 have varied significantly. For sensitivity (the rate at which a screening instrument correctly identifies a developmental delay) and specificity (the rate at which a screening instrument correctly identifies children who perform within the normal range), respectively, the following values have been reported: 75 and 86% [38]; 66 and 84% [14]; 82 and 78% [19]; 87.50 and 84.48% [45, 46]. Previous studies have also assessed the effectiveness of the ASQ-3 as a screening tool by comparing it with the Bayley-III and have found weak or moderate consistency between the two instruments [42, 49]. Some studies show that the ASQ-3 is better able to assess children’s development when children are older rather than when they are younger [31, 32, 40, 49]. The inconsistent results may be related to differences in reference measurement, age of the sample, cultural environments, and item explanations. Furthermore, measurement errors caused by the reference instrument itself may also be an explanation [42]. In spite of the discrepancies, a study by Wei et al. [45, 46] also pointed that ASQ-3 have good reliability and validity in mainland China, and can be used in the development screening and monitoring of eligible children in mainland China. Therefore, we also chose the ASQ-3 as a comparison scale.

The CREDI differs from the ASQ-3 as a population-level assessment tool because is not used to screen and diagnose an individual’s specific developmental problems or developmental delays, but provides caregivers with feedback on the child’s developmental status or tracks subtle changes in individual levels through intervention. This instrument is simpler and less burdensome to test, and parental involvement in testing may also help them gain important knowledge about their child’s development and understand their child’s performance at that age. It is an open resource that can be downloaded from the website https://sites.sph.harvard.edu/credi/ freely. It can serve to provide conceptually rich, developmentally informed, population-level data on global progress in alleviating ECD-related inequities and meeting target 4.2 of the UN Sustainable Development Goals [27].

The CREDI has been piloted and applied in many low- and middle-income countries around the world, and its reliability and validity have also been studied and analyzed. By analyzing 2481 caregivers of children aged 18–36 months in Tanzania, McCoy et al. [27] evaluated the acceptability, test-retest reliability, internal consistency, and discriminant validity of the newly developed CREDI items, subscales, and total scale. The results showed that the CREDI and its motor, cognitive, and social-emotional subscales had sufficient acceptability and internal consistency. It also found positive evidence for the validity of the CREDI by showing adequate criterion validity with the Bayley-III motor, cognitive, and communication subscales. The study also found that the CREDI can accurately distinguish differences in children’s ages, nutritional status, disabled status, and home stimulation activities. In addition to providing positive evidence of validity, the CREDI has been found to be a more acceptable tool in low-income environments because it is easily understood and quickly implemented, which was indicated by trained field staff with the equivalent of a secondary education level only spending about 20 min finishing the test on average. Moreover, it was found that this kind of caregiver-reported instrument is beneficial for reducing errors resulting from the non-compliance issues caused by factors such as unfamiliarity with the test environment, fear of unfamiliar adults, and children’s illnesses. However, coverage of the study in Tanzania was insufficient because it only included 18- to 36-month-old children, resulting in a lack of data regarding children younger than 18 months. The authors also suggest that before the CREDI is fully disseminated, more research in multilingual and cultural environments and lower age groups need to be conducted.

Another study about the application of the CREDI was also conducted by McCoy et al. [25] with 8022 participants from 17 low- and middle-income countries. The results showed that the CREDI short form is an effective, reliable, and acceptable population-level measure of ECD. Feedback from qualitative interviews with caregivers and field team members shows that participants have a good understanding of the CREDI, and it is easy to implement. Internal consistency was also sufficient. The results also show that the CREDI score differs among different social demographic subgroups. The criterion validity was also tested to be sufficient through the correlations between the CREDI and alternative ECD “gold standard” instruments. This study fully demonstrated that the CREDI short form is effective, reliable, and acceptable in measuring population-level ECD status in different cultural environments. Based on this, the authors suggest that the CREDI can be used as a useful tool to monitor ECD status in low-resource, low-cultural settings and in large-scale household surveys, while recommending the CREDI and other indicators be used together.

By using data from 1265 caregivers of infants and toddlers aged 0–35 months in Brazil, Altafim et al. [1] conducted a study to assess the acceptability, test-retest reliability, internal consistency, and discriminate and concurrent validity of the CREDI short form. The results of qualitative interviews showed that overall acceptance of the scale was high. Internal consistency was very high in the six age groups, with a coefficient greater than .8, but there were fewer participants in the 0–5 and 18–23 age groups, and therefore further research is required. Multivariate analysis of structural validity showed that some of the significant variations in CREDI scores could be explained by the child’s gender and family characteristics, such as women’s education levels, socioeconomic status, and stimulating activities of the family. Regarding concurrent validity, the CREDI score was significantly correlated with the PRIDI score, with a correlation coefficient of .46. In summary, the results of the study in Brazil show that the CREDI short form has high validity, reliability, and acceptability, which suggested that it can be used for assessment of ECD status on a large-scale in Brazil.

These studies conducted in-depth research on the application of the CREDI in low- and middle-income environments and mainly focus on the CREDI short form. Nevertheless, the reliability and validity of the CREDI long form in China has not yet been studied. The Chinese government’s recent commitment to the policy about childcare services urgently requires appropriate instruments to assess ECD status at the population level. Based on the previous review, the Bayley-III, although the gold standard, is not suitable for large-scale use because of its high cost of administering and administering requirements. Alternatively, although the ASQ-3 is suitable for large-scale use as a direct evaluation tool, there are still discrepancies in the data it provides. As an alternative measurement tool for the ASQ-3, the recently developed CREDI tool for the assessment of population-level ECD status in 17 low- and middle-income countries has been widely studied and recommended. However, before it is used in China, analysis of its reliability and validity is especially necessary. Therefore, the goal of this study was to evaluate the reliability and validity of the CREDI long form as a measure of ECD status at a population level in rural China. To do so, we administered the Bayley-III to a subsample of the total sample and administered the CREDI long form and ASQ-3 to their caregivers in the total sample. We then compared the outcomes of the CREDI test to those of the Bayley-III and the ASQ-3.

## Methods

### Data collection

The data used in this study were collected in a sample of 995 children aged 5–36 months from urban and rural communities in July 2018. It was based on data from a randomized controlled trial^1^ that required implemented intervention on children and their caregivers. However, in China, the traditional custom is that children from 0 to 5 months of age rarely go outside. Therefore, children in this age group were not the targeted group of the intervention and thus not in our study sample. The sample area is representative of one nationally-designated poor county in the Qinba mountain region of China. Each toddler’s primary caregiver was administered a detailed survey on parental and household characteristics, including each toddler’s age, gender, gestational age, presence of any siblings, whether the mother is the primary caregiver or not, maternal education, and household economic status.

All infants/toddlers were administered the two different scales to measure developmental outcomes: the CREDI long form and the ASQ-3. Considering this was the first application of the CREDI in China, the author’s research team engaged a professional translation company to conduct accurate translation and back-translation for the items and relative materials of the CREDI.^2^ The translation was welcomed by the CREDI team. The items translation was also sent to specialists in the field of child development for consultation and we conducted a pilot study in rural China before this survey to check whether the translation of the questions was clear and suitable for rural caregivers. Additionally, out of the 995 sample infants/toddlers, 258 were tested with the Bayley-III scales for their levels of cognitive, language, or motor development by enumeration teams.

It should be noted that the final sample for analysis is less than the full sample because of missing data. It comes from two sources: first, there is a very small proportion of missing values in key measures. We imposed strict quality control during data collection to avoid missing data and there were no missing data at the item level of the CREDI and Bayley-III, and less than 2% missing values at the item level of the ASQ-3. Second, there is less than 0.05% of child and family characteristics data missing in our sample, thus, we excluded this small proportion of missing sample in all analyses. Due to the small size of missing data and negligible effects on analysis, we excluded them from the analysis.

### Ethics

All study protocols were approved by institutional review boards (IRBs), both at Stanford University (No. 46564) and the West China School of Medicine, Sichuan University, China (No. K2018074). Caregivers provided written consent for their own participation and the participation of their children after a field worker read the consent form out loud and answered any questions. All study staff were trained and monitored in IRB-approved procedures for identifying participant needs.

### Measures

#### The Bayley scales of infant and toddler development, third edition (Bayley-III)

As one of the most widespread scales used to measure developmental status of infants and toddlers aged between 0 and 42 months, the Bayley-III is considered the gold standard in the field. The Bayley-III includes 326 items divided into five domains: cognition, receptive communication, expressive communication, fine motor, and gross motor. As each item is administered, the examiner records the child’s response and stops when there are five consecutive items wrong. The child gets 1 if he or she met the scoring criteria [3]; or else, the child gets 0. Then the sum of scaled scores for a given composite is calculated for each child in the normative sample from the Unites States. The scaled score with a mean of 10 and a standard deviation of 3 to composite score with a mean of 100 and a standard deviation of 15 equivalent is a linear conversion [4]. The Chinese version we used in the current study has been widely used in many researches on the early childhood development in China [23, 48–50]. The Chinese version is properly translated and back-translated by professional team.

#### The ages and stages questionnaire, third edition (ASQ-3)

The ASQ-3 is another widely used instrument to measure the developmental status of infants and toddlers aged between 1 and 66 months. The ASQ-3 includes 21 questionnaires, and each questionnaire consists of 30 simple, straightforward questions about five domains of childhood development: problem-solving, communication, gross motor, fine motor, and personal-social. The answer to each question is selected from three possible responses: “yes,” “sometimes,” or “not yet.” Caregivers should select “yes” if the child shows a specific behavior, “sometimes” if the specific behavior is occasional or new, or “not yet” if the question refers to a behavior the child has not yet shown. The total score of each domain is determined by calculating the score of six questions in each domain. By comparing the total score of the five domains with the threshold value (It equals the mean value minus 2 standard deviations of each domain) of the corresponding domain obtained by empirical research, the development status of the child can be determined. The Chinese version we used in the study is validated by Bian et al. [5] and Wei et al. [45, 46].

#### Caregiver reported early childhood development instruments (CREDI)

Newly developed scales used to measure ECD status at the population level, the CREDI,^3^ aim to provide an accurate and easy-to-administer assessment of ECD for children between 0 and 35 months that functions across a wide variety of cultural, linguistic, and socioeconomic contexts [1, 25]. CREDI is directly tested by the child’s primary caregiver using a scale that is answered “yes” or “no” (If caregivers are unsure of their response, they may also choose to respond by saying “don’t know”). The CREDI team also set up the *credi* package in the software program R to guide users scoring the CREDI long form [26].

As part of a larger project, both a short and a long form of the CREDI were developed from the same broad item set. The long form produces a score for each of the domains, namely cognitive, language, motor, and social-emotional developmental status. The goal of the long form is to provide detailed information to researchers interested in measuring specific developmental domains. The long form consists of a total of 108 items, and the starting point is determined according to the child’s age, and the ending point is determined according to a five-link error/uncertainty factor. In contrast, the short form can produce a total score for the child’s overall developmental status, which contains 20 items selected to characterize children’s development within predefined six-month age bands. For research and evaluation projects, the long form will provide domain-specific details about the child development, which can capture differences in the specific skills that help the design of the intervention targeted to improve child development. In the current study, therefore, the long form was used and evaluated.

In sum, all of the tests can capture the children’s developmental status for each domain. The age range covered in each test is different. The age period of the CREDI is the shortest, from 0 to 35 months; the age period of the Bayley-III is moderate, from 0 to 42 months; the age period of the ASQ-3 is the longest, from 1 to 66 months. The CREDI can cover younger children, while the ASQ-3 can cover older children compared to the CREDI and Bayley. In terms of administration, compared to the Bayley-III, both the CREDI and the ASQ-3 are shorter in duration and easier to administer. Actually, during our survey, the CREDI was very simple and clear enough to be answered by a caregiver with minimal formal education. The cost of administration was also lower than the Bayley-III.

### Statistical analysis strategy

First, the descriptive characteristics of the sample were displayed to show the basic information about the corresponding sample and the ECD status measured by different instruments. Second, reliability was assessed with the items internal consistency and Cronbach’s α coefficients were used to interpret the internal consistency of these three questionnaires. Next, internally standardized correlations among these measures were calculated to test concurrent validity. At the same time, to obtain the heterogeneous analysis results, the samples were divided into age cohorts of 5–11 months, 12–17 months, 18–23 months, 24–29 months, and 30–35 months when calculating the correlations. The samples were also divided according to the type of caregiver and the household wealth status, and the correlations calculated. Finally, an ordinary least squares (OLS) regression was conducted to check the relationship between a set of variables shown to be related to child development and the scores on these three instruments. This analysis tested whether the three measures have consistent predictive factors or not. It is one way to determine the similarity of the three measures in terms of how they identify developmental status of the children. All statistical analyses were performed using Stata 14.2 statistical software.

## Results

First, the descriptive characteristics of the sample are displayed in Table 1. As shown in Table 1, 946 toddlers were included in our final analysis. Among these toddlers, only 248 were administered the Bayley-III. The distribution of child and family characteristics between the total sample and Bayley sample were mostly consistent. Generally, there were a slightly higher proportion of female toddlers; slightly over half had siblings; around 5% of the sample were born prematurely; the mother was identified as the primary caregiver for about 70% of the toddlers; the educational attainment of mothers was low overall – around half had junior high school and below. The household wealth status was moderate among all samples, and the wealth status of the Bayley sample was relatively better than the total sample.

**Table 1:** Descriptive characteristics of the sample

Child and family characteristics	Total sample		Bayley sample	
	N	%	N	%
Age group				
5–11 months	191	20.19	50	20.16
12–17 months	215	22.73	56	22.58
18–23 months	186	19.66	52	20.97
24–29 months	206	21.78	62	25.00
30–35 months	148	15.64	28	11.29
Gender				
Female	487	51.48	131	52.82
Male	459	48.52	117	47.18
Sibling				
No	495	52.33	125	50.40
Yes	451	47.67	123	49.60
Premature (gestational age < 37 weeks)				
Yes	44	4.65	12	4.84
No	902	95.35	236	95.16
Primary caregiver				
Mother	661	69.87	172	69.35
Others	285	30.13	76	30.65
Mother’s education				
Junior high school and below	520	54.97	120	48.39
Senior high school and above	426	45.03	128	51.61
Household wealth index, mean (SD)	946	0.01(0.87)	248	0.24(0.78)
Home stimulation				
Participated in fewer than four of these six activities	636	67.23	158	63.71
Participated in at least four of these six activities	310	32.77	90	36.29

Note: In China, junior high school refers to chu zhong (初中; 初级中学; literally low-level middle school) from grades 7 to 9; senior high school refers to gao zhong (高中; 高级中学; literally high-level middle school) from grades 10 to 12. The household wealth factor was constructed to indicate the wealth status of the sample. The factor was based on the following nine variables: annual agricultural and non-agricultural income in 2017, and seven dummy variables of being registered as a poor household, having a flush toilet, water heater, PC, internet access, air conditioning, and a truck/car at home. It was generated using the iterated principal-factor method to calculate factor loadings and derive a factor score for each household. Questions about home stimulation activities in our survey asked whether, in the past 3 days, the primary caregiver had engaged their children in any of the following activities: reading books or looking at picture-books, telling stories, singing songs, taking child outside home for playing, playing with the child with toys, spending time with the child in naming things, counting, or drawing. Referred to the clarification about this variable from Multiple Indicator Survey by UNICEF [10], it is treated as a binary variable here

Second, the ECD results are shown in Table 2. The mean scores (SD) from the CREDI indicated that the overall developmental status of our sample was only moderate. In terms of the Bayley-III scores, the Bayley-III has not yet been administered to a healthy reference population in China. As such, we rely on reference populations from other widely accepted research, which reveals that, for a healthy population, the mean score (SD) is expected to be 105 (9.6) for the cognitive scale [20, 33], 109 (12.3) for the language score [33], and 107 (14) for the motor score [7, 20]. According to the above standards, the developmental status of our sample was slightly below average. With respect to the ASQ-3, the mean scores of each domain were a little lower than the referenced mean scores shown in the ASQ-3 user guide. In sum, the results obtained from the three tests were generally consistent with slightly better results from the CREDI.

**Table 2:** The summary statistics of the CREDI, Bayley-III, and ASQ-3

	Mean	SD	Min	Max
**1. CREDI(*n*** **= 946)**				
**Z-score**				
Cognitive	0.058	1.095	−2.981	5.887
Language	0.334	1.068	−2.682	6.951
Motor	−0.012	1.020	−3.311	6.069
Social-emotional	0.287	1.047	−3.010	5.101
**2. Bayley-III(*n*** **= 248)**				
**Composite scores**				
Cognitive	102.036	13.527	65.000	145.000
Language	103.423	16.911	62.000	153.000
Motor	105.871	16.436	61.000	151.000
**3. ASQ-3(*n*** **= 955)**				
**Scores**				
Problem Solving	46.549	12.500	0.000	60.000
Communication	45.327	13.604	0.000	60.000
Fine Motor	47.611	13.937	0.000	60.000
Gross Motor	41.897	14.621	0.000	60.000
Personal-Social	44.904	11.644	0.000	60.000

Note: The CREDI Z-scores were constructed by comparing the raw score in each domain to the average raw score in our CREDI reference population of a particular age. A z-score of 0 thus means that the child has exactly the same score on that particular domain as the average same-age child in the CREDI reference population. A score of “-1” means that the child’s raw score is 1 standard deviation below the same-age average of the reference population (more details about the reference population can be found in the CREDI data management & scoring manual). The range score for each domain of Bayley-III composite scores is 55 to 145 for Cognitive, 47 to 153 for Language, and 46 to 154 for Motor, respectively. The raw score range of the ASQ-3 for each domain is from 0 to 60

Third, the internal consistency of the CREDI, Bayley-III, and ASQ-3 are shown in Table 3. Both the CREDI and Bayley-III have large Cronbach’s α coefficients, which means the internal consistency of the two scales was high. For the CREDI, the Cronbach’s α coefficients of each subscale ranged from .92 to .97. When the internal consistency was examined by age group, it was found that the Cronbach’s α coefficients of each subscale decreased accordingly, but remained relatively high. For age 6–11 months, the Cronbach’s α coefficients of each subscale ranged from .81 to .87; for age 12–17 months, it ranged from .83 to .91; for age 18–23 months, it ranged from .74 to .93; for age 24–29 months, it ranged from .66 to .91; for age 30–35 months, it ranged from .60 to .89. Overall, the Cronbach’s α coefficients of the cognitive, motor, and social-emotional subscales decreased with age, but increased before 12 months. For the language subscale, the Cronbach’s α coefficients decreased with age, but increased before 24 months. Besides, it should be noted that the CREDI has unacceptably low internal consistency reliability (Cronbach’s α coefficients are below .7) in some places, such as the motor and social-emotional subscale within age 24–35 months.

**Table 3:** Internal consistency of the CREDI, Bayley-III, and ASQ-3 (*Overall and by age groups*)

Test	Scales	Cronbach’s alpha					
		Total	6–11 months	12–17 months	18–23 months	24–29 months	30–35 months
CREDI	Cognitive	0.9395	0.8498	0.8632	0.8154	0.8227	0.7634
	Language	0.9693	0.8720	0.9057	0.9287	0.9121	0.8886
	Motor	0.9374	0.8085	0.8258	0.7440	0.6844	0.6606
	Social-emotional	0.9178	0.8213	0.8624	0.7977	0.6546	0.6040
Bayley-III	Cognitive	0.9773	0.9701	0.9718	0.9738	0.9454	0.8752
	Expressive communication	0.9722	0.9650	0.9708	0.9654	0.9450	0.9027
	Receptive communication	0.9720	0.9703	0.9712	0.9638	0.9466	0.9256
	Fine motor	0.9685	0.9645	0.9767	0.9803	0.9415	0.8641
	Gross motor	0.9724	0.9648	0.9606	0.9456	0.8989	0.7931
ASQ-3	Problem Solving	0.5450	0.5945	0.7075	0.3741	0.4183	0.6397
	Communication	0.6314	0.4898	0.4886	0.7164	0.6684	0.5210
	Fine Motor	0.6081	0.6196	0.6512	0.4847	0.6034	0.6635
	Gross Motor	0.6963	0.5896	0.8040	0.5078	0.5555	0.4951
	Personal-Social	0.4084	0.5134	0.5145	0.3487	0.4786	0.4246

Note: In our case, we treated the items as binary items in the analysis, thus we follow Hill & Lewicki [13]‘s suggestion which stated that “Cronbach’s alpha, when computed for binary (e.g., true/false) items, is identical to the so-called Kuder-Richardson-20 formula of reliability for sum scales”

For the Bayley-III, the Cronbach’s α coefficients of each subscale ranged from .97 to .98. The Cronbach’s α coefficients of each subscale decreased after the sample was divided by age group. Despite this, the internal consistency reliability indicated by the Cronbach coefficients was still very high. For the subscales of cognitive and fine motor skills, the internal consistency reliability increased with age between 6 and 23 months, while the internal consistency reliability decreased with age after 23 months. For the subscales of receptive communication and expressive communication, the internal consistency reliability increased with age between 6 and 17 months, while it decreased with age after 17 months. For the subscale of gross motor, the internal consistency reliability decreased with age across the five age groups.

In contrast, the ASQ-3 had a relatively lower scale internal consistency reliability. The Cronbach’s α coefficients of each subscale ranged from .41 to .70. Among the five subscales, the internal consistency reliability of the gross motor subscale was the highest and its Cronbach coefficient was .70; the internal consistency reliability of the personal-social subscale was the lowest and its Cronbach coefficient was .41. When the sample was divided by age group, the Cronbach’s α coefficients varied irregularly within different age groups.

Subsequently, the correlations between the CREDI and Bayley-III scores, the ASQ-3 and Bayley-III scores, and the CREDI and the ASQ-3 scores for each of their domains by age group were calculated respectively. *P*-values of the correlations were calculated by bootstrapping methods, with 1000 replications. As shown in Table 4, the results indicated that the concurrent validity of the CREDI with the Bayley-III scale was high in general. That is, CREDI cognitive, language, and motor subscales had strong correlations with the corresponding Bayley-III subscales. The correlation coefficients ranged from .84 to .90, among which the correlation between the CREDI motor subscale and the Bayley-III gross motor subscale was the largest. In contrast, although the correlation coefficients between the ASQ-3 communication subscale and the Bayley-III expressive communication and receptive communication subscales, and the ASQ-3 gross motor subscale and the Bayley-III gross motor subscale were significant at moderate levels, the concurrent validity of the ASQ-3 with the Bayley-III scale was relatively lower in general. With respect to the concurrent validity of the ASQ-3 with the CREDI, the results showed that only the correlation coefficients between the ASQ-3 communication subscale and the CREDI language subscale, as well as the ASQ-3 gross motor subscale and the CREDI motor subscale were significant at moderate levels, and the correlations in other domains were extremely weak.

**Table 4:** Correlations among CREDI, Bayley-III, and the ASQ-3

	Bayley III (***n*** = 248)								
	Cognitive	RC	EC	Fine Motor	Gross Motor				
**CREDI**									
Cognitive	0.835***	0.791***	0.814***	0.828***	0.864***				
Language	0.889***	0.855***	0.897***	0.870***	0.852***				
Motor	0.879***	0.813***	0.839***	0.872***	0.901***				
Social-emotional	0.868***	0.803***	0.831***	0.856***	0.896***	**CREDI (*n*** **= 946)**			
**ASQ-3**						Cognitive	Language	Motor	Social-emotional
Problem Solving	−0.100+	− 0.017	− 0.053	−0.069	− 0.117*	0.116***	0.060+	0.059+	0.059+
Communication	0.319***	0.404***	0.426***	0.322***	0.249***	0.388***	0.472***	0.348***	0.362***
Fine Motor	−0.181**	−0.134*	− 0.145*	− 0.167**	− 0.171**	0.060+	− 0.016	0.009	− 0.002
Gross Motor	0.429***	0.373***	0.391***	0.426***	0.549***	0.491***	0.442***	0.533***	0.484***
Personal-Social	−0.036	0.047	0.031	−0.011	0.016	0.196***	0.133***	0.153***	0.149***

*** *p* < 0.001, ** *p* < 0.01, *p < 0.05, + *p* < 0.1

Note: Pearson correlations on internally standardized scores. Since raw scores are increasing in age, we eliminated the age effect by internally standardizing raw scores within age (month) groups. This is done by computing age-adjusted z-scores using age-conditional means and standard deviations estimated by non-parametric regression. Compared to parametric procedures, the advantage of this non-parametric standardization method is less sensitive to outliers and small sample size within age category and yields normally distributed standardized scores with mean zero across the age range (in months) ([2] [22];). Standard Errors (SE) computed using bootstrap method. Bootstrapping allows estimation of the sampling distribution of almost any statistic using random sampling methods. Bootstrap is asymptotically more accurate than the standard intervals obtained using sample variance and assumptions of normality. *RC* Receptive Communication, *EC* Expressive Communication

The heterogeneous analysis of the CREDI was also conducted, as shown in Tables 5, 6 and 7. The correlations were calculated among the Bayley-III, the CREDI, and the ASQ-3 by age group, primary caregiver, and wealth status. Table 5 shows that the correlations between the CREDI and Bayley-III varied with different age groups. In general, the correlation between the CREDI and the Bayley-III was strong before 18 months but was relatively weak at 18 to 23 months, and was moderate after 24 months. When the correlations between the ASQ-3 and Bayley-III by age group were examined, it was found that, generally, the correlation in the domain of communication between ASQ-3 and Bayley-III was better within 12–29 months than other age periods. With respect to the correlations between the CREDI and the ASQ-3 by age group, it was found that within 5–11 months, only the correlation between the CREDI language subscale and the ASQ-3 communication was significant and at a moderate level. After 12 months, the correlations between each domain of the CREDI and the ASQ-3 were significant and moderate.

**Table 5:** Correlations among CREDI, Bayley-III, and the ASQ-3 by Age Group

	Bayley III, 5–11 months(***n*** = 50)								
	Cognitive	RC	EC	Fine Motor	Gross Motor				
**CREDI**									
Cognitive	0.386**	0.374**	0.515***	0.600***	0.566***				
Language	0.449***	0.447**	0.508***	0.559***	0.515***				
Motor	0.472***	0.424**	0.585***	0.634***	0.617***				
Social-emotional	0.450***	0.413**	0.544***	0.606***	0.602***	**CREDI, 5–11 months(*n*** **= 191)**			
**ASQ-3**						Cognitive	Language	Motor	Social-emotional
Problem Solving	−0.007	−0.182	− 0.079	− 0.105	−0.100	0.078	0.029	0.104+	0.032
Communication	0.117	0.255+	0.179	0.162	0.104	0.322***	0.422***	0.253***	0.339***
Fine Motor	0.038	−0.122	−0.021	− 0.069	− 0.131	0.032	0.046	0.012	0.009
Gross Motor	−0.062	0.004	−0.056	−0.121	0.017	0.047	0.004	0.047	0.028
Personal-Social	0.121	0.100	0.052	−0.027	0.067	0.159*	0.126+	0.123+	0.130+
	**Bayley III, 12–17 months(*n*** **= 56)**								
	Cognitive	RC	EC	Fine Motor	Gross Motor				
**CREDI**									
Cognitive	0.460***	0.516***	0.512***	0.505***	0.624***				
Language	0.399**	0.559***	0.644 ***	0.528***	0.514***				
Motor	0.442***	0.529***	0.527***	0.532***	0.736***				
Social-emotional	0.480***	0.488***	0.508***	0.497***	0.631***	**CREDI, 12–17 months(*n*** **= 215)**			
**ASQ-3**						Cognitive	Language	Motor	Social-emotional
Problem Solving	0.073	0.319**	0.286**	0.422**	0.095	0.429***	0.381***	0.354***	0.427***
Communication	0.128	0.403***	0.440***	0.409**	0.082	0.241***	0.348***	0.126+	0.228**
Fine Motor	0.082	0.208+	0.224*	0.207	0.070	0.430***	0.346***	0.400***	0.415***
Gross Motor	0.335**	0.188	0.170	0.304*	0.695***	0.400***	0.293***	0.570***	0.437***
Personal-Social	0.352*	0.328**	0.327**	0.427***	0.348**	0.496***	0.410***	0.399***	0.513***
	**Bayley III, 18–23 months(*n*** **= 52)**								
	Cognitive	RC	EC	Fine Motor	Gross Motor				
**CREDI**									
Cognitive	0.450***	0.254*	0.329***	−0.021	0.201				
Language	0.539***	0.381***	0.546***	0.134	0.245+				
Motor	0.498***	0.262*	0.275**	0.003	0.181				
Social-emotional	0.379***	0.260*	0.294**	0.007	0.200	**CREDI, 18–23 months(*n*** **= 186)**			
**ASQ-3**						Cognitive	Language	Motor	Social-emotional
Problem Solving	0.023	−0.030	0.063	0.089	−0.094	0.341***	0.298***	0.389***	0.302***
Communication	0.428***	0.395***	0.551***	0.105	0.243+	0.583***	0.758***	0.572***	0.568***
Fine Motor	0.062	−0.041	−0.024	− 0.080	− 0.139	0.408***	0.276***	0.415***	0.338***
Gross Motor	0.044	0.014	0.112	0.070	0.157	0.178*	0.149*	0.337***	0.127
Personal-Social	−0.086	0.083	0.211+	−0.027	− 0.118	0.256**	0.197*	0.306***	0.227**
	**Bayley III, 24–29 months(*n*** **= 62)**								
	Cognitive	RC	EC	Fine Motor	Gross Motor				
**CREDI**									
Cognitive	0.228+	0.463***	0.419***	0.343**	0.284*				
Language	0.369**	0.534***	0.612***	0.386***	0.194				
Motor	0.095	0.183	0.190	0.253*	0.370**				
Social-emotional	0.187	0.360**	0.317*	0.315**	0.281*	**CREDI, 24–29 months(*n*** **= 206)**			
**ASQ-3**						Cognitive	Language	Motor	Social-emotional
Problem Solving	0.126	0.208+	0.092	0.080	0.200	0.491***	0.399***	0.370***	0.444***
Communication	0.202+	0.410***	0.455***	0.290*	0.343*	0.467***	0.604***	0.404***	0.410***
Fine Motor	−0.010	0.116	0.128	0.097	0.238+	0.451***	0.329***	0.401***	0.366***
Gross Motor	−0.216*	−0.153+	− 0.198*	−0.095	0.091	0.271***	0.154*	0.351***	0.191**
Personal-Social	−0.031	0.129	0.213+	0.110	0.347**	0.520***	0.515***	0.528***	0.475***
	**Bayley III, 30–35 months(*n*** **= 28)**								
	Cognitive	RC	EC	Fine Motor	Gross Motor				
**CREDI**									
Cognitive	0.230	0.228	0.205	0.259	0.157				
Language	0.554***	0.550***	0.560**	0.575***	0.309*				
Motor	0.250	0.267+	0.263	0.426***	0.242				
Social-emotional	0.246	0.162	0.181	0.196	0.033	**CREDI, 30–35 months(*n*** **= 148)**			
**ASQ-3**						Cognitive	Language	Motor	Social-emotional
Problem Solving	0.121	0.219	0.012	0.019	−0.025	0.455***	0.453***	0.411***	0.371***
Communication	0.388*	0.399**	0.288	0.138	0.210	0.475***	0.480***	0.356***	0.431***
Fine Motor	0.306+	0.240	0.140	0.243	0.235	0.452***	0.340***	0.451***	0.401***
Gross Motor	0.476**	0.258	0.470*	0.401*	0.248	0.377***	0.379***	0.466***	0.243***
Personal-Social	0.073	0.359**	0.015	0.132	0.040	0.484***	0.394***	0.543***	0.443***

For the description of asterisks, please refer to Table 4 footer

**Table 6:** Correlations among CREDI, Bayley-III, and the ASQ-3 by caregiver

	Bayley III, Mother is the primary caregiver (***n*** = 172)								
	Cognitive	RC	EC	Fine Motor	Gross Motor				
**CREDI**									
Cognitive	0.850***	0.811***	0.839***	0.841***	0.885***				
Language	0.900***	0.875***	0.910***	0.889***	0.874***				
Motor	0.879***	0.813***	0.833***	0.873***	0.902***				
Social-emotional	0.880***	0.821***	0.849***	0.868***	0.915***	**CREDI(*n*** **= 661)**			
**ASQ-3**						Cognitive	Language	Motor	Social-emotional
Problem Solving	−0.088	0.001	−0.026	−0.042	− 0.092	0.117**	0.073+	0.069+	0.069+
Communication	0.287***	0.404***	0.425***	0.295***	0.251***	0.379***	0.458***	0.346***	0.349***
Fine Motor	−0.243**	−0.197**	− 0.197**	− 0.196**	− 0.184**	0.020	− 0.056	−0.022	− 0.032
Gross Motor	0.408***	0.357***	0.368***	0.386***	0.528***	0.528***	0.488***	0.563***	0.526***
Personal-Social	−0.089	−0.002	−0.013	− 0.069	−0.020	0.153***	0.095*	0.109**	0.106**
	**Bayley III, Grandmother is the primary caregiver (*n*** **= 66)**								
	Cognitive	RC	EC	Fine Motor	Gross Motor				
**CREDI**									
Cognitive	0.800***	0.752***	0.744***	0.813***	0.822***				
Language	0.869***	0.822***	0.869***	0.840***	0.807***				
Motor	0.874***	0.812***	0.848***	0.868***	0.895***				
Social-emotional	0.830***	0.763***	0.779***	0.836***	0.851***	**CREDI(*n*** **= 251)**			
**ASQ-3**						Cognitive	Language	Motor	Social-emotional
Problem Solving	−0.127	− 0.054	− 0.109	− 0.120	−0.175+	0.169*	0.072	0.086	0.090
Communication	0.379***	0.400***	0.437***	0.397***	0.252*	0.436***	0.543***	0.379***	0.427***
Fine Motor	−0.004	0.031	0.003	−0.060	− 0.099	0.213**	0.132+	0.154*	0.134*
Gross Motor	0.445***	0.382***	0.404***	0.486***	0.577***	0.365***	0.286***	0.418***	0.332***
Personal-Social	0.021	0.104	0.072	0.067	0.043	0.296***	0.205**	0.255***	0.249***

For the description of asterisks, please refer to Table 4 footer

**Table 7:** Correlations among CREDI, Bayley-III, and the ASQ-3 by wealth status

	Bayley III, 25% poorest (***n*** = 38)								
	Cognitive	RC	EC	Fine Motor	Gross Motor				
**CREDI**									
Cognitive	0.792***	0.722***	0.717***	0.781***	0.823***				
Language	0.902***	0.821***	0.867***	0.869***	0.848***				
Motor	0.846***	0.764***	0.781***	0.869***	0.878***				
Social-emotional	0.841***	0.768***	0.779***	0.829***	0.869***	**CREDI(*n*** **= 235)**			
**ASQ-3**						Cognitive	Language	Motor	Social-emotional
Problem Solving	−0.094	− 0.084	− 0.162	0.089	− 0.072	0.157*	0.091	0.112	0.112
Communication	0.357**	0.389**	0.306*	0.298*	0.329*	0.409***	0.491***	0.367***	0.398***
Fine Motor	−0.317*	− 0.318*	− 0.337*	− 0.207	−0.154	− 0.019	−0.087	− 0.046	−0.076
Gross Motor	0.389*	0.338*	0.336+	0.488***	0.594***	0.442***	0.359***	0.475***	0.420***
Personal-Social	−0.009	0.103	0.050	0.115	0.040	0.220**	0.140*	0.167*	0.183*
	**Bayley III, 25% richest (*n*** **= 79)**								
	Cognitive	RC	EC	Fine Motor	Gross Motor				
**CREDI**									
Cognitive	0.802***	0.833***	0.841***	0.800***	0.851***				
Language	0.873***	0.905***	0.921***	0.869***	0.835***				
Motor	0.883***	0.866***	0.882***	0.865***	0.910***				
Social-emotional	0.845***	0.847***	0.858***	0.833***	0.884***	**CREDI(*n*** **= 235)**			
**ASQ-3**						Cognitive	Language	Motor	Social-emotional
Problem Solving	−0.023	0.091	0.093	0.004	−0.044	0.105*	0.055	0.048	0.046
Communication	0.337***	0.455***	0.521***	0.437***	0.274**	0.352***	0.470***	0.345***	0.322***
Fine Motor	−0.182	−0.091	− 0.056	− 0.155	−0.098	0.056	−0.014	− 0.014	−0.004
Gross Motor	0.400***	0.371***	0.412***	0.414***	0.580***	0.430***	0.385***	0.505***	0.435***
Personal-Social	−0.067	0.033	0.065	0.002	0.070	0.110+	0.050	0.071	0.064

For the description of asterisks, please refer to Table 4 footer

When the correlations among the three tests were examined by caregiver type and household wealth status, it was found that, in general, regardless of whether the primary caregiver was the mother or the grandmother, or whether the household wealth status was poor or rich, the correlations between the CREDI and Bayley-III were large and statistically significant. The correlations between ASQ-3 and Bayley III were only significant and moderate in the domains of communication and gross motor, and the correlations between the CREDI and ASQ-3 were significant but relatively small. This is shown in Tables 6 and 7.

To complete the analysis, the OLS regression results were reviewed to check whether the three instruments have consistent predictors. All the scores received from the Bayley-III, CREDI, and ASQ-3 were internally standardized before OLS regression.

As shown in Appendix Table, children from homes with higher stimulation obtained higher Bayley cognitive scores; the older the children, the higher the Bayley cognitive scores. When the same factors were used to predict children’s CREDI cognitive scores, some consistencies with the Bayley-III results were evident. That is, the higher the home stimulation, the higher the CREDI cognitive scores; and the CREDI cognitive scores increased with the child’s age. However, different from the Bayley cognitive, household wealth status was positively related to the CREDI cognitive with a very small effect (indicated by the small coefficient). The child’s gender was negatively related to the CREDI cognitive, that is, girls had higher CREDI scores than boys. Additionally, when the same factors were used to predict children’s ASQ-3 scores, in a similar way to the CREDI and Bayley III, home stimulation was positively related to ASQ-3 “Problem Solving”. Consistent with the CREDI while inconsistent with the Bayley-III, household wealth status was positively related to the ASQ-3. Different from the other two tests, type of primary caregiver was significantly related to the ASQ-3. When the primary caregiver was the mother, the ASQ-3 “Problem Solving” score was higher.

When the same factors were used to predict children’s language scores, it was found that children with higher home stimulation obtained higher Bayley “Receptive Communication” and “Expressive Communication” scores; the older the children, the higher the Bayley scores; girls’ Bayley scores were higher than boys’ s. When the same factors were used to predict children’s CREDI language scores, the results were consistent with the Bayley-III. However, different from the Bayley-III, household wealth status was positively related to CREDI language scores, while the correlation between household wealth status and Bayley III “Receptive communication” and “Expressive communication” was insignificant. When the same factors were used to predict children’s ASQ-3 communication scores, the results were a little different. Just as with the CREDI and Bayley-III, home stimulation was positively related to ASQ-3 “Communication”, and girls obtained higher scores than boys. Consistent with the CREDI while inconsistent with the Bayley III, household wealth status was positively related to ASQ-3. Different from the other two tests, the relationship between age and the ASQ-3 communication score varied with age group. Compared to age 5–11 months, only children aged 18 months and above were higher in ASQ-3 “Communication”.

When the same factors were used to predict children’s motor scores, the results were both consistent as well as inconsistent among the three instruments. Specifically, motor development measured by the three instruments was positively related to children’s age. With respect to the predictor “home stimulation”, the correlation between home stimulation and Bayley motor was insignificant, but home stimulation was positively related to CREDI motor and ASQ motor. The child’s gender was significantly related to CREDI motor rather than Bayley motor and ASQ-3 motor. Whether the child was premature or not was significantly related to the Bayley fine motor results, rather than the CREDI motor and ASQ-3 motor.

In terms of predicting the development of children’s social emotional data, only the ASQ-3 “Personal-Social” and CREDI “Social-Emotional” were assessed because of the lack of a Bayley-III “Social-Emotional” category in our study. The results showed that both the ASQ-3 and CREDI scores were positively related to home stimulation, and girls obtained higher social-emotional scores than boys.

Above all, there was high consistency in predicting ECD status among the three tests in some key predictors, such as home stimulation. That is, higher Bayley scores (except Bayley motor scores), CREDI scores, and ASQ-3 scores were positively related to home stimulation. There were also some consistent results in some individual and family characteristics. For example, the results showed the scores of the three tests indicating language and social emotional development level were higher for girls than boys. Nevertheless, there were inconsistent results in predicting ECD status in some individual and family characteristics. For example, children’s gender was not significantly related to Bayley-III cognitive and motor scores, but was closely related to CREDI scores. Household wealth status was not significantly related to Bayley-III scores, but was positively related to CREDI and ASQ-3 scores indicating cognitive and language development level. With respect to caregiver type, the results of Bayley-III and CREDI scores suggested there was no significant association. However, the results for ASQ-3 scores indicated the caregiver type was connected to the ASQ-3 “Problem- Solving” scores.

In general, the results showed relatively consistent predictors of scores about the level of ECD through different measurements. It can be concluded that as a caregiver-reported, population-level measurement for children’s development, the CREDI is highly consistent with previous widely used instruments in some key predictors (such as home stimulation) concerning the ECD level. Moreover, the CREDI is highly consistent with indirect assessment, namely the ASQ-3, in some individual and family characteristics (such as the children’s gender and household wealth status).

## Discussion

From the above information, it can be concluded that the administration time, difficulty, and cost of the CREDI is more advantageous than the Bayley-III, and the internal consistency reliability and validity of the CREDI is also more advantageous than another indirect measurement, that is, the ASQ-3.

First, according to the results shown above, the Bayley scales have very good internal consistency, whereas the ASQ-3 has unacceptably poor internal consistency reliability. In contrast, the Cronbach’s α coefficients of each CREDI subscale were large, despite declining when the sample was divided by age group, which indicates the internal consistency reliability of the CREDI was still good in general. However, it should be noted the CREDI has unacceptably low internal consistency reliability in the motor and social-emotional subscale within age 24–35 months. Second, concurrent validity analysis conducted using the Bayley-III as the criterion indicated generally high concurrent validity of the CREDI. In contrast, the concurrent validity of the ASQ-3 with the Bayley-III scale was low, and the concurrent validity of the two indirect assessments, the CREDI and the ASQ-3, was also low. Third, heterogeneous analysis generally showed that the correlation between the CREDI and the Bayley-III was strong before 18 months but relatively weak at 18–23 months, and was moderate after 24 months. In contrast, the correlation in the domain of communication between ASQ-3 and Bayley-III was better within 12–29 months than other age periods. In terms of the correlation between the CREDI and ASQ-3 by age group, within 5–11 months, only the correlation between the CREDI language subscale and ASQ-3 communication was significant and at a moderate level. After 12 months, the correlations between each domain of the CREDI and the ASQ-3 were significant and moderate. In addition, the heterogeneous analysis showed that there are no big differences in the correlations between the CREDI and Bayley-III by caregiver types and household wealth statuses. Finally, OLS analysis showed that, the CREDI was highly consistent with previous widely-used instruments in some key predictors (such as home stimulation) of ECD level. Furthermore, the CREDI was also highly consistent with the indirect assessment, namely the ASQ-3, in some individual and family characteristics (such as the children’s gender and household wealth status).

Compared with previous studies, the current study examined the reliability and validity of the CREDI long form in China, which hasn’t been assessed before. Moreover, the coverage under 3 years of age is extensive and each age group are included except for 0–5 months. Consistent with previous research, the results in the current study suggested that the CREDI can be used as a useful tool to monitor ECD status in impoverished regions of China at large scale. Multivariate regression results are consistent with previous study that emphasizes the importance of home stimulation activities and family economic status. At present, the development of childcare services under 3 years old in China is lagging behind. Systematic and effective childcare policies and services have not yet been formed, and the absence of supportive systems and the shortage of social services are prominent [47]. Especially after the implementation of the universal two-child policy, the establishment of the childcare policy system has attracted unprecedented attention. The information about the ECD status at the population level is the basis for the Chinese government in the implementation of childcare policies and services and the development of more effective intervention strategies. The current study makes implications for the use of the CREDI long form to monitor the ECD outcomes in impoverished regions of China. Besides, this study also indicates that the public services and support provided by the society cannot completely replace the function of the family, and the improvement of family members’ parenting practices (reflected by the home stimulation activities) is conducive to effectively improving the early development of children in poor rural areas. Despite its merits, the current study has several limitations. First, there are some limitations on using the motor and social-emotional subscale with children aged 24–35 months. The possible reason needs to be explored further. Second, there was an issue with collection of concurrent gold-standard measures of child development with which to determine the CREDI’s concurrent validity. Concurrent validity with direct observation, that is, using the Bayley-III, was tested only for just over two hundred children. Because of the small sample, which lacked corresponding representation, the conclusion of the current study cannot be generalized to the whole Shaanxi Province or China. The focus on a single geographic context for the sample also limits the generalizability of these results. Besides, the measure invariance is not assessed at this stage. Future studies should include samples from geographically, linguistically, developmentally, and culturally diverse contexts of China. Third, there was a lack of inter-rater reliability for the study coordinators who administered the CREDI. Although the two local study coordinators who administered the items of the screening tool by verbal interview were fluent in Mandarin, possible communication issues or varying levels of comprehension should be considered, particularly given the wide range in education backgrounds of the caregivers. Lacking of the test-retest reliability was also a limitation of the study, which should be done in our future studies. At last, our lack of a “gold standard” metric against which to compare our social-emotional items limits our understanding of their concurrent validity in the current study. Children aged 0–5 months old were not included in the study, which makes it impossible to verify and analyze the reliability and validity of the scale for children aged 0–5 months.

## Conclusion

Providing high-quality ECD services in low- and middle-income countries would require joint efforts from all sectors, effective management, adequate funding, an ample workforce, community and parental collaboration, reliable data systems, continuous monitoring, and evaluation and improvement cycles [28]. It can be concluded from the current study that the CREDI is a feasible low-cost instrument for use in large-scale data collection for early developmental intervention. In China, due to the long-term urban-rural dual economic system and economic development gap, it has been found that there are significant urban-rural differences in early childhood development [18]. As a feasible population-level measurement of ECD, the use of the CREDI long form in China is beneficial to improve ECD outcomes and reduce developmental inequality through national, and regional policies and resource allocation. However, it should be noted the CREDI long Form still lacks the ability to provide information about individual children. It may also not be sensitive enough to detect smaller changes attributable to intervention. The CREDI team also pointed out that in spite of the value of the CREDI long form in intervention evaluation, a more detailed and domain-focused measure should be paired with whenever possible (Please refer to the CREDI User Guide). Besides, given the CREDI is a caregiver-reported scale, using a direct assessment (such as Bayley) as the triangulation of measurement is useful to address potential weakness in one approach versus another. Therefore, there is much more work that needs to be done in the future so that the instrument can be effectively used for population level monitoring and research purposes. The use of the CREDI long form to assess the interventions effects in China also should be evaluated in the future studies.

## Data Availability

The data used in the current study is not publicly available because its confidentiality. However, it is available from the corresponding author on reasonable request.

## Appendix

**Table 8:** Results of multivariate regression analysis predicting scores related with the level of early childhood development

Variables	Bayley Cog	CREDI Cog	ASQ-3 SP	Bayley RC	Bayley EC	CREDI Lang	ASQ-3 Com	Bayley GM	Bayley FM	CREDI Mot	ASQ-3 GM	ASQ-3 FM	CREDI SE	ASQ-3 PS
Home stimulation	0.116*(0.054)	0.375*** (0.040)	0.430*** (0.083)	0.305*** (0.074)	0.296*** (0.061)	0.298*** (0.039)	0.389*** (0.059)	0.067 (0.049)	0.068 (0.060)	0.229*** (0.045)	0.242*** (0.055)	0.527*** (0.082)	0.277*** (0.032)	0.326*** (0.068)
Age group (Reference: [5]-11months)														
12–17 months	1.005*** (0.103)	1.155*** (0.062)	−0.165 (0.130)	0.717*** (0.069)	0.808*** (0.087)	0.824*** (0.051)	−0.312*** (0.087)	1.524*** (0.098)	1.005*** (0.124)	1.122*** (0.050)	0.865*** (0.110)	−0.293** (0.100)	1.188*** (0.056)	0.028 (0.108)
18–23 months	1.571*** (0.098)	1.701*** (0.057)	−0.146 (0.112)	1.394*** (0.116)	1.418*** (0.088)	1.500*** (0.045)	−0.089 (0.109)	1.957*** (0.069)	1.470*** (0.117)	1.759*** (0.047)	1.203*** (0.092)	−0.231* (0.097)	1.806*** (0.047)	0.055 (0.108)
24–29 months	2.293*** (0.107)	2.026*** (0.056)	−0.368*** (0.105)	1.949*** (0.111)	2.152*** (0.088)	2.098*** (0.040)	0.468*** (0.085)	2.406*** (0.065)	2.202*** (0.120)	2.163*** (0.045)	1.073*** (0.075)	−0.467*** (0.096)	2.179*** (0.046)	−0.215* (0.101)
30–35 months	2.737*** (0.136)	2.233*** (0.070)	−0.350** (0.125)	2.316*** (0.188)	2.349*** (0.151)	2.381*** (0.046)	0.521*** (0.101)	2.582*** (0.121)	2.665*** (0.172)	2.491*** (0.057)	1.154*** (0.094)	−0.673*** (0.145)	2.391*** (0.068)	−0.058 (0.121)
Gender (Reference: Female)														
Male	−0.051 (0.050)	−0.133*** (0.032)	− 0.053 (0.065)	−0.214** (0.064)	− 0.192** (0.071)	−0.134*** (0.026)	− 0.209** (0.069)	−0.021 (0.053)	− 0.099 (0.074)	−0.097*** (0.027)	0.025 (0.053)	−0.076 (0.063)	− 0.095** (0.029)	−0.299*** (0.059)
With sibling or not (Reference: no sibling)														
With sibling	−0.037 (0.058)	−0.030 (0.038)	− 0.085 (0.057)	−0.011 (0.052)	− 0.025 (0.079)	−0.033 (0.030)	− 0.021 (0.053)	−0.017 (0.049)	− 0.044 (0.060)	−0.036 (0.033)	− 0.015 (0.060)	−0.019 (0.068)	0.001 (0.036)	−0.069 (0.055)
Whether premature or not (Reference: No)														
Yes	−0.188+ (0.099)	−0.021 (0.083)	0.064 (0.197)	−0.130 (0.186)	0.045 (0.111)	−0.032 (0.078)	− 0.041 (0.176)	− 0.055 (0.123)	− 0.283* (0.129)	− 0.077 (0.072)	0.160 (0.112)	0.033 (0.126)	− 0.038 (0.071)	0.110 (0.188)
Whether mother is the primary caregiver (Reference: No)														
Yes	0.005 (0.062)	−0.039 (0.038)	0.162* (0.075)	−0.098 (0.100)	−0.118 (0.088)	− 0.024 (0.035)	0.040 (0.073)	− 0.038 (0.052)	−0.095 (0.065)	− 0.002 (0.029)	−0.022 (0.064)	0.075 (0.064)	−0.061+ (0.034)	0.029 (0.081)
Whether the mother’s education level is high school and above (Reference: No)														
Yes	0.013 (0.048)	0.033 (0.041)	0.067 (0.083)	0.061 (0.071)	0.013 (0.057)	0.019 (0.034)	0.106 (0.072)	0.013 (0.048)	0.033 (0.041)	0.067 (0.083)	0.061 (0.071)	0.013 (0.057)	0.019 (0.034)	0.106 (0.072)
Household wealth indicator	0.073	0.045+	0.144**	0.106	0.047	0.051*	0.117*	0.011	0.028	0.024	0.080*	0.062	0.033	0.073+
	(0.049)	(0.025)	(0.043)	(0.070)	(0.057)	(0.023)	(0.046)	(0.036)	(0.044)	(0.019)	(0.037)	(0.039)	(0.023)	(0.038)
Constant	−1.196*** (0.145)	−1.387*** (0.169)	0.086 (0.211)	−0.994*** (0.221)	−1.319*** (0.168)	− 1.317*** (0.136)	−0.260 (0.219)	−1.488*** (0.122)	− 1.245*** (0.182)	−1.377*** (0.114)	− 0.872*** (0.212)	0.393 (0.294)	− 1.471*** (0.184)	0.311 (0.308)
Observations	248	946	946	248	248	946	946	248	248	946	946	946	946	946
R-squared	0.884	0.736	0.163	0.796	0.825	0.807	0.253	0.875	0.843	0.818	0.287	0.169	0.802	0.131

Note: *** *p* < 0.001, ** *p* < 0.01, * *p* < 0.05, + *p* < 0.1. All standard errors were clustered at village level. Numbers in parentheses are standard errors. The examiner’s ids were also controlled in all regression results. All scores are internally standardized. *Bayley Cog* Bayley Cognitive, *Bayley RC and Bayley EC* Bayley Receptive Communication and Bayley Expressive Communication, *Bayley GM* Bayley Gross Motor, *Bayley FM* Bayley Fine Motor, *CREDI Cog* CREDI Cognitive, *CREDI Lang* CREDI Language, *CREDI Mot* CREDI Motor, *CREDI SE* CREDI Social-emotional, *ASQ SP* ASQ Problem Solving, *ASQ-3 Com* ASQ-3 Communication, *ASQ-3 GM* ASQ-3 Gross Motor, *ASQ-3 FM* ASQ-3 Fine Motor, *ASQ PS* ASQ Personal-social

## Abbreviations

ECD: Early Childhood Development
CREDI: Caregiver Reported Early Childhood Development Instruments
ASQ-3: The Ages and Stages Questionnaire, third edition
Bayley-III: The Bayley Scales of Infant and Toddler Development, third edition

## Footnotes

1 Trial registration: ISRCTN, ISRCTN16736104. Registered 25 May 2018, 10.1186/ISRCTN16736104

2 According to the CREDI’s user guide, cultural adaptation of the CREDI is not required. Nevertheless, appropriate translation of the CREDI is critical to its utility and comparability as a population-level measure.

3 A thorough discussion of the instrument’s construction (i.e., item construction, IRT testing, etc.) as well as the psychometrics of the CREDI short form is provided by McCoy et al. [25]. Details regarding the items developed and the psychometrics of the CREDI long form are introduced in the forthcoming paper by Waldman et al. [44], which is conducted by the CREDI Team in the School of Public Health, Harvard University, and reveals that the CREDI’s motor, cognitive, language, and socio-emotional subscales developed using a multidimensional framework exhibited adequate internal-consistency and test-retest reliability, as well as sufficient concurrent validity evidence.
